# A diagnostic dilemma: autoimmune pulmonary alveolar Proteinosis unmasked by vaping-induced lung injury

**DOI:** 10.1093/omcr/omag190

**Published:** 2026-10-05

**Authors:** Mohammed Najdat Seijari, Taraneh Zamani, Mohammad Nasser Affas, Souwdamini Sethuram, Marwa Alfaraj, Muhammad Umair Khawar

**Affiliations:** Department of Internal Medicine, TriHealth, Good Samaritan Hospital, 375 Dixmyth Avenue, Cincinnati, OH 45220, United States; Department of Internal Medicine, TriHealth, Good Samaritan Hospital, 375 Dixmyth Avenue, Cincinnati, OH 45220, United States; Department of Internal Medicine, Hamad Medical Corporation, Hamad Medical City, Doha, Qatar; Department of Internal Medicine, TriHealth, Good Samaritan Hospital, 375 Dixmyth Avenue, Cincinnati, OH 45220, United States; Faculty of Medicine, Damascus University, Al-Mazzeh Street, Damascus, Syria; Department of Pulmonary and Critical Care Medicine, TriHealth, Good Samaritan Hospital, 375 Dixmyth Avenue, Cincinnati, OH 45220, United States

**Keywords:** pulmonary alveolar Proteinosis, vaping-induced lung injury, diffuse alveolar damage

## Abstract

A 65-year-old smoker with recent vaping resumption, found to have elevated anti-GM-CSF antibodies suggestive of autoimmune pulmonary alveolar proteinosis (PAP). However, bronchoalveolar lavage and whole lung lavage were inconclusive, raising suspicion for concurrent E-cigarettes or vaping-associated lung injury (EVALI). The patient showed no clinical response to corticosteroids and eventually passed away. Post-mortem pathology confirmed surfactant accumulation and foamy macrophages but also showed features of diffuse alveolar damage. This case underscores the diagnostic and management challenges when PAP and inhalational lung injuries overlap.

## Introduction

Pulmonary Alveolar Proteinosis (PAP) is a surfactant homeostasis disorder that presents on a spectrum of severity from mild disease with spontaneous clearance to acute lung injury that can be fatal [1]. It is classified into three major types: autoimmune (primary or idiopathic), secondary, and congenital. Autoimmune PAP results from autoantibodies against GM-CSF, impairing surfactant clearance by alveolar macrophages, while congenital PAP results from defects in GM-CSF signaling or receptors [1].

Secondary causes of PAP often involve diseases that impair macrophage surfactant clearance, such as myelodysplastic syndrome, leukemias, immunodeficiency syndromes, autoimmune diseases, and exposure to inhaled fumes or industrial dust like silica or titanium [2].

Overall, more than 90% of PAP cases are autoimmune, with the rest being a mix of secondary, congenital, and PAP-mimickers [2]. Epidemiologic studies estimating a prevalence of approximately 6–7 cases per million population and an annual incidence of less than 1 case per million [3]. A large Japanese cohort estimated a minimum incidence and prevalence of 0.49 and 6.2 cases per million, respectively, while a large U.S. claims-based study reported a prevalence of 6.87 cases per million [4].

## Case presentation

A 65-year-old female with a medical history including chronic obstructive pulmonary disease (COPD), daily tobacco smoking, recent resumption of vaping (having previously quit 7 years ago), gastroesophageal reflux disease (GERD), presented with a one-month history of progressive shortness of breath. She had no relevant occupational or environmental exposures, medication-related pulmonary risk factors, diabetes, or other known causes of immunocompromise. Her symptoms began with cough and wheezing, for which she received outpatient treatment for presumed COPD exacerbation, including two steroid courses—the second combined with doxycycline for 10 days—without clinical improvement. She subsequently developed fever, chills, and generalized weakness. At presentation, she was in hypoxemic respiratory failure requiring 2 L nasal cannula oxygen. Her white blood cell count was elevated (18000), but procalcitonin was negative. Viral testing, including COVID-19 and influenza, was negative. On examination, she exhibited diffuse crackles at the lung bases with minimal late expiratory wheezing and poor air movement. A CT scan of the chest revealed extensive bilateral ground-glass opacities (Fig. 1). Multiple differentials based on her imaging and rapid clinical deterioration were entertained, including PAP. Therefore, serologic workup including an autoimmune panel and GM-CSF antibodies, was sent.

**Figure 1:**
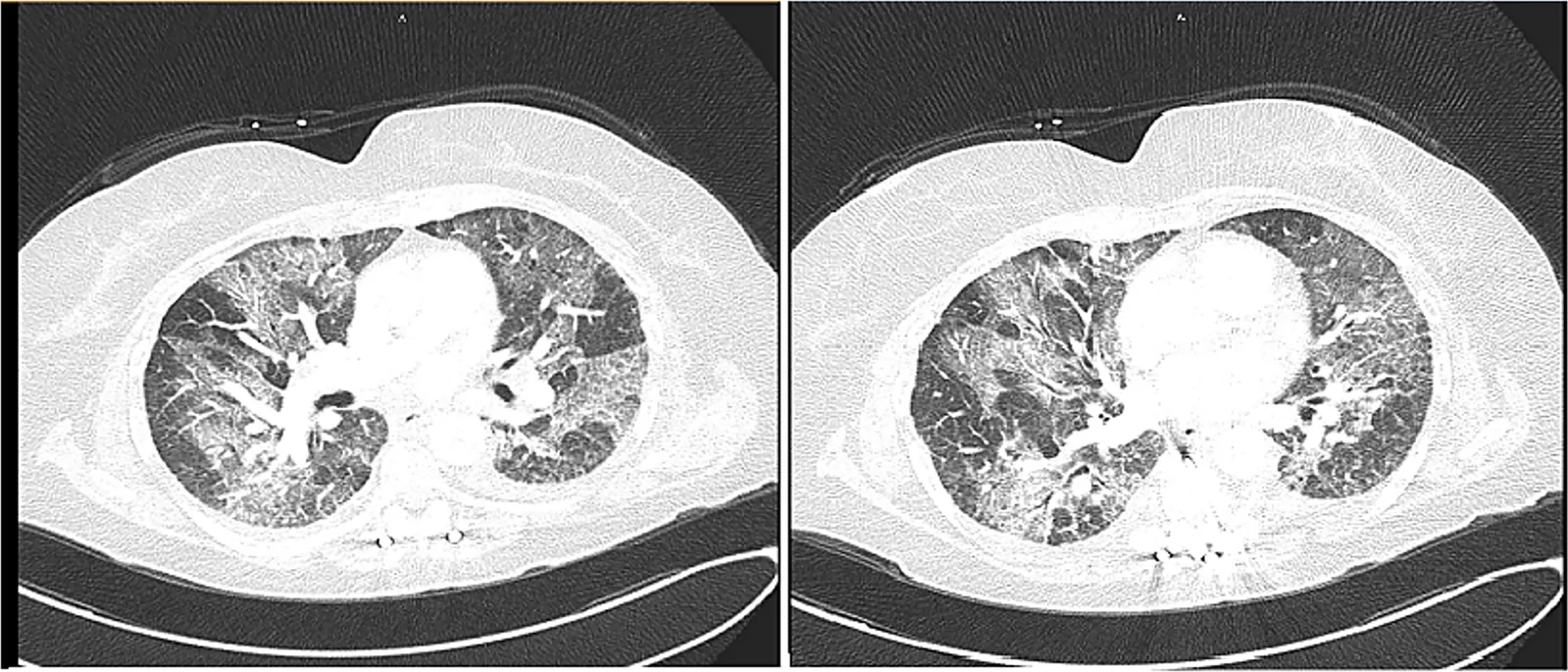
CT chest, day 1 (middle and lower cuts).

She received IV furosemide, nebulizers, and empiric antibiotics (ceftriaxone, azithromycin). Bronchoalveolar lavage (BAL) on day 3 showed 44% neutrophils, 43% macrophages; fungal smear, cytology, and cultures were all negative. There was no evidence of alveolar hemorrhage or acute eosinophilic pneumonia, and the fluid lacked the characteristic opaque appearance seen in PAP. She was then started on high-dose IV methylprednisolone, PJP prophylaxis, and piperacillin-tazobactam for 7 days as her clinical picture worsened.

The patient’s respiratory condition worsened, necessitating increased support with high-flow oxygen, CPAP, intubation, mechanical ventilation, and eventual transfer to the ICU. Serial CT chest imaging indicated that the initial findings persisted despite treatment (Fig. 2). Despite interventions such as proning and paralysis, her condition worsened. Testing revealed elevated GM-CSF autoantibodies, suggesting autoimmune PAP, but repeated BAL and imaging were not definitive. Additional tests (including AFB smear, fungal markers, anti-GBM, PJP, ANA, ANCA, and peripheral smear) were negative (Table 1). She was transferred for refractory hypoxemia management and underwent whole lung lavage, which did not show classic findings of PAP. Periodic acid–Schiff stain (PAS) stain was negative. She required extracorporeal membrane oxygenation (ECMO), developed bloodstream infections from central lines and ECMO, and received antibiotics and antifungals. Following a tracheostomy and persistent hypoxemia, her condition continued to decline; after multidisciplinary discussion, comfort care was chosen and she passed away.

**Figure 2:**
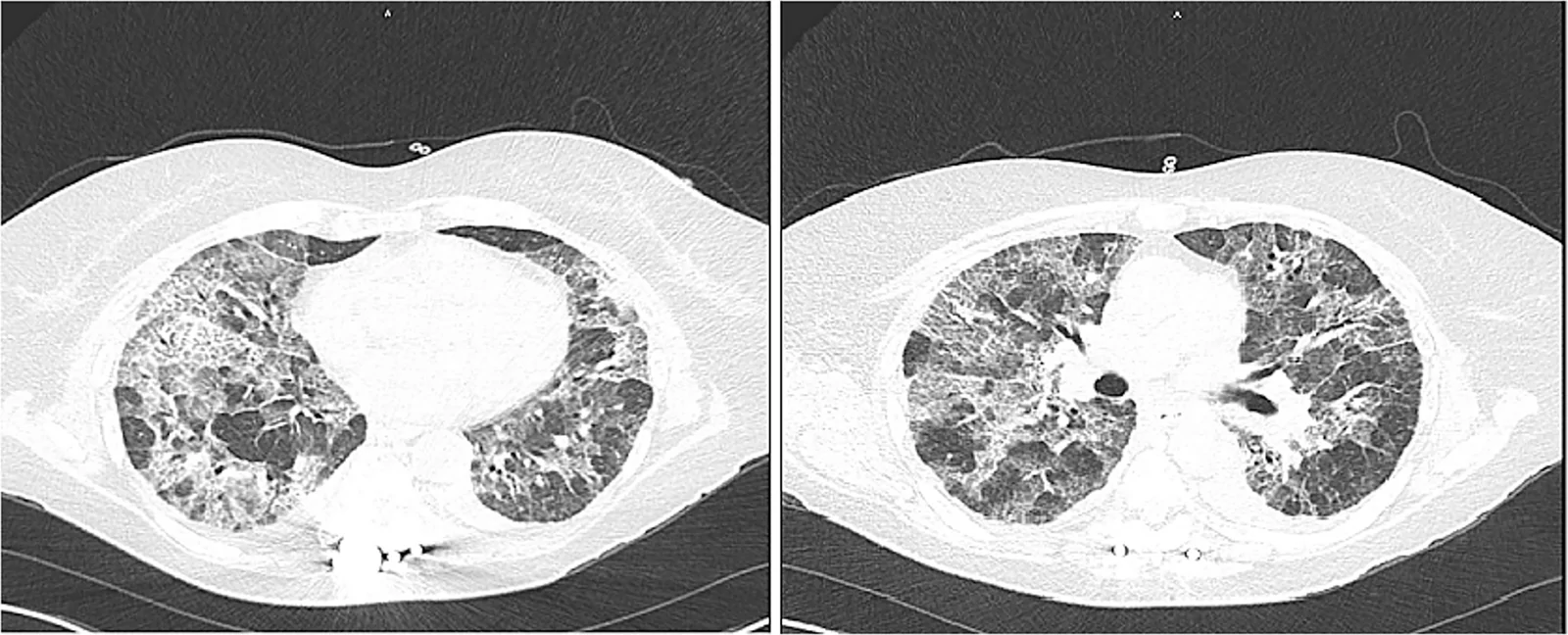
CT chest, day 8 (middle and lower cuts).

**Table 1:** Laboratory test results.

Test	Result (units)	Reference Range
GM-CSF Autoantibody	610	< 338 ng/ml
ANCA (C-ANCA)	< 1:20	Normal
ANCA (P-ANCA)	< 1:20	
ANA by IFA	Negative	
SS-A/Ro AB	< 0.2	
SS-B/La AB	< 1/2	
JO-1 AB	< 0.2	
Rheumatoid Factor	<10	
CMV DNA Quantitative	Not Detected	
SARS-CoV-2 PCR (NAA)	Negative	Nasopharyngeal swab
COVID-19 Antigen (Rapid)	Negative	
CRP (C-Reactive Protein)	58 mg/L	< 5 mg/L
HIV 1/2 AB	Nonreactive	
IgG	525	586–1602 IU/ml
IgA	239	90–410 IU/ml
IgM	54	30–360 IU/ml
IgE	29	6–495 IU/ml
Influenza A PCR	Negative	
Influenza B PCR	Negative	
MRSA PCR	Negative	
Fungitell 1,3 beta-D glucan	< 31 (negative)	
Legionella antigen (urine)	negative	
Histoplasma antigen	Negative	
Pneumocystis jirovecii PCR	Negative	
AFB TB DNA PCR	Not Detected	
ARP/70 Antibodies	Not Detected	
Anti-PR3 Antibodies	Not Detected	
Aspergillus AG	Not Detected	
CENTROMERE AB SCREEN	Not Detected	
Coccidioides AG IgG/IgM/Serum	Not Detected	
DS DNA Fungal Panel Molecular	Not Detected	
Histoplasma Blastomyces Ag Result/M Ab Yeast	Negative	
SCL-70 AB	<0.2	
SM AB	<0.2	
SM/RNP AB	<0.2	

A post-mortem lung examination was performed. Microscopic examination showed that there was widespread interstitial lymphocytic infiltrate, while the alveolar spaces had hyaline membranes and intra-alveolar proteinaceous material that was PAS positive. Macrophages in proteinaceous areas were vacuolated and ‘foamy,’ while other areas demonstrated pigment-laden ‘smoker’s macrophages’. Special stains for HSV, CMV, GMS, adenovirus, and acid-fast bacilli were all negative. The etiology of her acute lung injury remained multifactorial, with possible contributions from vaping-related lung injury causing a secondary PAP.

## Discussion

Diagnosing PAP is challenging and requires ruling out similar conditions. Assessment includes detailed history, focusing on occupational exposure and signs of hematological or autoimmune diseases, evaluated through history and serology. Chest CT typically reveals diffuse ground glass opacities, patchy geographic patterns, and occasionally a crazy paving appearance—the latter being rare in secondary PAP. Pulmonary function tests may show reduced carbon monoxide diffusion, though this finding is not specific to PAP [5].

Bronchoalveolar lavage (BAL) is a key tool for diagnosing PAP, typically showing milky fluid and PAS-positive proteinaceous material with few inflammatory cells. Combined with clinical, radiological evidence—like crazy-paving on CT—and anti-GM-CSF antibody tests for autoimmune PAP, these findings are usually diagnostic. While transbronchial lung biopsy (TBLB) is more invasive, it is useful when BAL results are inconclusive or atypical; other options include video-assisted thoracoscopic surgery (VATS) and open lung biopsy [6].

The presence of antibodies against GM-CSF is highly sensitive and specific (100% and 98% respectively) [7] and is a helpful non-invasive method in diagnosing PAP.

PAP treatment typically includes supplementation with GM-CSF (commonly administered via inhalation), anti-CD20 therapy, plasmapheresis, and whole lung lavage (WLL). WLL is considered the standard procedure for severe cases with significant hypoxemia; it is performed on each lung separately under anesthesia and may be repeated as needed until improvement is observed. Lung transplantation is an option for cases that do not respond to other treatments [8].

Active Smoking was reported in patients with PAP to be around 79% and interestingly, it carried an increase for treatment resistance, as smokers on average needed in this cohort 5 WLL (whole lung lavage) compared to an average of 2.4 in non-smokers [9]. Therefore, recent vaping may have contributed to an overlapping acute lung injury and potentially complicated the clinical course and response to whole-lung lavage. Animal studies on EVALI indicate that smoking and vapor inhalation increase proinflammatory cytokines, such as GM-CSF, in the lungs. Currently, there is no definitive evidence connecting vaping to the development of anti-GM-CSF antibodies [10]. Additional research is needed to assess the impact of inhalation injury on surfactant homeostasis and its potential role in triggering autoimmune PAP.

## Novelty and distinction from previously reported cases

Although autoimmune PAP associated with vaping has previously been reported, including a case with positive serum anti-GM-CSF antibodies and vitamin E detected in BAL fluid [11], our case differs in its fulminant course and atypical diagnostic findings. Diagnosis was challenging because BAL lacked the characteristic milky appearance, initial PAS staining was negative, and whole-lung lavage neither demonstrated classic PAP findings nor produced clinical improvement. Post-mortem examination ultimately demonstrated extensive intra-alveolar PAS-positive proteinaceous material and foamy macrophages, supporting PAP in conjunction with positive anti-GM-CSF autoantibodies. Secondary PAP following EVALI has also been reported, with PAS-positive material and ultrastructural surfactant abnormalities [12]. However, unlike that patient, who responded favorably to corticosteroid therapy without requiring whole-lung lavage, our patient showed no clinical response to corticosteroids and progressed to refractory hypoxemic respiratory failure requiring ECMO, ultimately resulting in death.

## Conclusion

This case highlights the diagnostic challenges of PAP, especially when autoimmune markers are present but bronchoscopy and lavage are inconclusive. Smoking and vaping may hinder treatment and cause overlapping lung injuries. A multidisciplinary approach is necessary for accurate interpretation of findings. The possible link between vaping-related lung injury and surfactant disorders merits further study.
